# Evolution from Natural *β*-Carboline Alkaloids to Obtain 1,2,4,9-tetrahydro-3-thia-9-aza-fluorene Derivatives as Potent Fungicidal Agents against *Rhizoctonia solani*

**DOI:** 10.3390/ijms19124044

**Published:** 2018-12-14

**Authors:** Junmin Xi, Zhijun Zhang, Qi Zhu, Guohua Zhong

**Affiliations:** Key Laboratory of Crop Integrated Pest Management in South China, Ministry of Agriculture, South China Agricultural University, Guangzhou 510642, China; xijunmin1990@163.com (J.X.); zhangzhijun198803@163.com (Z.Z.); zhuqi1008611@163.com (Q.Z.)

**Keywords:** *β*-carboline, 1,2,4,9-tetrahydro-3-thia-9-azafluorene, fungicidal activity, *Rhizoctonia solani*

## Abstract

Rice sheath blight, caused by *Rhizoctonia solani*, is a globally important rice disease and the increasing resistance of this pathogen highlights the need for new active compounds against rice sheath blight. In this study, natural *β*-carboline alkaloids were optimized to obtain a series of 1,2,4,9-tetrahydro-3-thia-9-aza-fluorene derivatives and evaluated for their fungicidal activity and mode of action against *R. solani*. Of these compounds, **18** exhibited significant in vitro fungicidal activity against *R. solani*, with an EC_50_ value of 2.35 μg/mL, and was more active than validamycin A. In vivo bioassay also demonstrated that **18** displayed superior protective and curative activities as compared to validamycin A. Mechanistically, **18** not only induced the loss of mitochondrial membrane potential and accumulation of reactive oxygen species, but also interfered with DNA synthesis. Therefore, compound **18** displayed pronounced in vitro and in vivo fungicidal activity against *R. solani* and could be used as a potential candidate for the control of rice sheath blight.

## 1. Introduction

Rice sheath blight, caused by *Rhizoctonia solani* [1], is one of the most important and destructive fungal diseases in rice around the world [2], and has caused devastating losses of yield up to 50% under favorable conditions as well as decreased quality of rice [3,4]. Although a few fungicides are available and have been widely used for the control of rice sheath blight [5], such as validamycin A [6], indiscriminate fungicide usage not only increases the risk of pathogen resistance but also harms the environment and human health [7]. Therefore, the quest for highly-effective fungicides against rice sheath blight with a novel mode of action is imperative.

**Figure 1:**
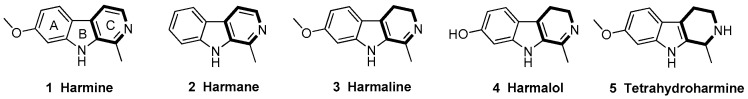
Representative structures of the *β*-carboline alkaloids.

Natural bioactive products are considered as ideal lead compounds to develop biorational alternatives compared to synthetic agrochemicals [8,9]. *β*-Carboline alkaloids and the saturated analogues, dihydro-*β*-carboline and tetrahydro-*β*-carboline alkaloids, are the main active components from medicinal genus *Peganum*, such as *Peganum harmala*, *P. multisectum*, and *P. nigellastrum*, which are distributed in the northwest of China [10]. Harmine (1), harmane (2), harmaline (3), harmalol (4), and tetrahydroharmine (5) are the representative harmala alkaloids (Figure 1), and these alkaloids have demonstrated marginal and similar fungicidal activity against several pathogenic fungi including *R. solani* [11,12], which implies that ring C of *β*-carboline is attractive for further optimization. In our previous studies, we introduced the functional groups urea [13] and oxadiazole [14] into the three position of *β*-carboline and showed an enhancing fungicidal activity against *R. solani*. However, the inflexible property of the *β*-carboline scaffold led to the poor solubility of those *β*-carboline derivatives. Thus, in this work, we are focused on the modification of ring C of the *β*-carboline scaffold, including changing the degrees of saturation and introducing heteroatoms, and providing a potent 1,2,4,9-tetrahydro-3-thia-9-aza-fluorene scaffold which can be used as a novel fungicidal template against *R. solani*. Then, a series of 1,2,4,9-tetrahydro-3-thia-9-aza-fluorene derivatives are synthesized and systematically evaluated for their in vitro and in vivo fungicidal activities against *R. solani*. Meanwhile, the preliminary mechanism of action are also elucidated.

## 2. Results and Discussion

### 2.1. Chemistry

**Figure 2:**
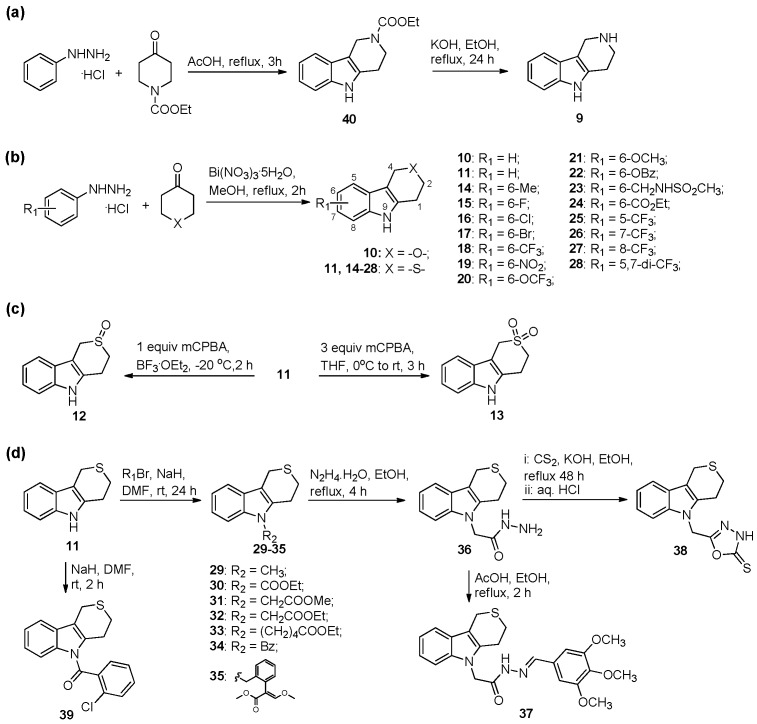
Synthesis of target compounds **9**–**39**.

The synthesis of intermediates and target compounds were performed as shown in Figure 2. Following a Fischer indole synthesis protocol, various phenylhydrazine hydrochlorides were reacted with cycloalkanone under acidic condition or bismuth–nitrate catalyst to afford compounds **9**–**11** and **14**–**28** [15,16]. Oxidation of **11** with different ratios of m-chloroperbenzoic acid gave oxide **12** and dioxide **13** [15]. N-substituted compounds **29**–**35** were achieved in a good yield through coupling of **11** and various alkyl halides or acyl chloride under alkaline conditions [15]. Treatment of **32** with hydrazine hydrate in ethanol obtained acylhydrazone **36**, which converted into compound **37** on treatment with 3,4,5-trimethoxybenzaldehyde [17], and into oxadiazole **38** according to our previously reported procedure [14]. *N*-benzoylation of **11** was accomplished using 2-chlorobenzoyl chloride in the presence of sodium hydride, furnishing compound **39**. All newly synthesized compounds were purified by column chromatography and their structures were confirmed on the basis of ^1^H NMR (nuclear magnetic resonance) and elemental analysis data.

### 2.2. Biological Activity

**Figure 3:**
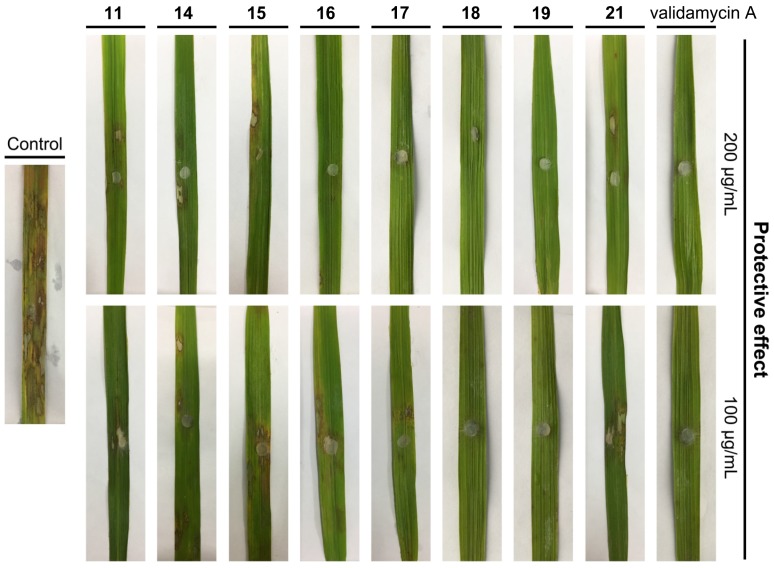
The in vivo protective effect of selected compounds against *R. solani* using detached leaf assay.

**Figure 4:**
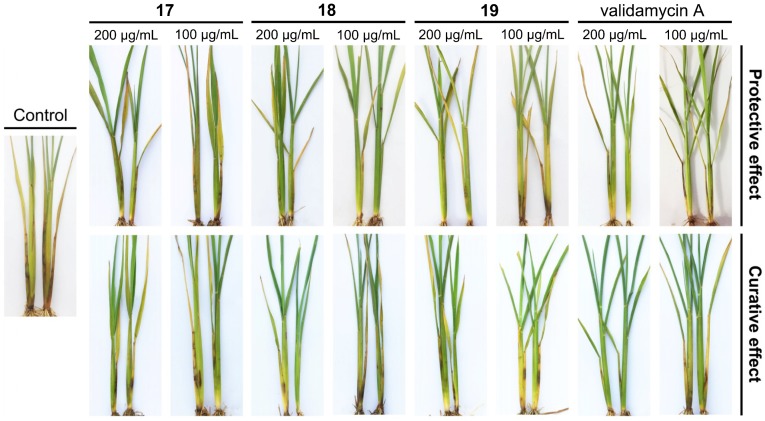
The in vivo protective and curative activities of compounds **17**–**19** against *R. solani.*

The results of the in vitro and in vivo fungicidal activities of compounds **6**–**39** against *R. solani* are listed in Table 1, Table 2, Table 3, Table 4 and Figure 3 and Figure 4 with commercial fungicide validamycin A as a positive control.

#### 2.2.1. In Vitro Fungicidal Activity of Compounds **6**–**39** against *R. solani*

Harmine (**1**) and its analogs **3** and **5**–**13** were screened for their fungicidal activity against *R. solani* and the results presented in Table 1 show that these three *β*-carboline alkaloids displayed similar fungicidal activity against *R. solani*, indicating that the C ring of *β*-carboline is attractive for further optimization. For example, the activities of harmine (**1**), harmaline (**3**), and tetrahydroharmine (**5**) were 30.25%, 29.51%, and 28.31%, respectively, at 100 μg/mL. Therefore, the initial round of structure activity relationship (SAR) modifications was directed toward optimization for the C ring while maintaining the tricyclic core scaffold. First, the phenyl group (**7**) was substituted for the pyridine moiety (**6**) and led to a slight improvement in activity, whereas when the C ring was tetrahydropyridine, compound **8** was inactive. Interestingly, tetrahydro-*γ*-carboline (**9**) was approximately 10-fold more active than the *β*-carboline counterpart **8**. Second, the influence of the heteroatoms on the C ring was explored; this led to the synthesis of compounds **10** and **11**, which were favorable for activity. Of particular note, the hit compound **11** displayed the most activity in the series with an EC_50_ (medium effective concentration) value of 38.58 μg/mL and was 8-fold more potent than **1** (EC_50_ = 318.56 μg/mL). Oxidation of compound **11** obtained sulfoxide **12** and sulfone **13**, which were bereft of activity (EC_50_ > 1000 μg/mL). These results indicated that 1,2,4,9-tetrahydro-3-thia-9-aza-fluorene was a potent fungicidal scaffold.

The effects of the substituents and substituted position on the A ring of 1,2,4,9-tetrahydro-3-thia-9-aza-fluorene were subsequently investigated. As shown in Table 2, small groups (**15**–**20**) were favorable for activity, while bulky groups (**22**,**23**) were detrimental to activity. Compounds **15**–**20** bearing electron-withdrawing groups were more potent than **11**, whereas electron-donating groups (**14**,**21**) were significantly less active. For example, compounds **17**–**19** showed the best potency with respective EC_50_ values of 2.94, 2.35, and 3.79 μg/mL. For the effect of the substituted position on the ring A, the fungicidal activity of compounds (**18**,**25**–**27**) increased following 6-CF_3_ > 5-CF_3_ > 8-CF_3_ > 7-CF_3_. In comparison to monosubstituted compounds **25** and **26**, disubstituted compound **28** was much less potent. Finally, the biological data for **29**–**39** with a focus on varying substituents was in the 9-position of the 1,2,4,9-tetrahydro-3-thia-9-aza-fluorene moiety. Here, it can be seen quite clearly that the structural changes in general were detrimental to activity and no favorable substitution could be found for the 9-position, even after extensive probing with a variety of diverse groups (**29–39**).

#### 2.2.2. In Vivo Protective and Curative Effects against Rice Sheath Blight

Among the compounds tested for fungicidal activity in vitro, compounds **11**, **14**–**19**, and **21** were potent against *R. solani* and, therefore, were selected to evaluate the in vivo protective effect using detached leaf assay. As shown in Table 3 and Figure 3, most of these compounds exhibited excellent in vivo protective potency. Fortunately, compounds **17**–**19** possessed the highest fungicidal activity and the inhibitory effect at 100 μg/mL was up to 97.41%, 99.87% and 100%, respectively, which was comparable in activity to the commercial fungicide validamycin A (100% at 100 μg/mL).

The most potent compounds **17**–**19** were further assessed for their protective and curative effects against *R. solani* under a greenhouse experiment, and the results were presented in Table 4 and Figure 4. All of three compounds displayed significant in vivo fungicidal activity and effectively controlled rice sheath blight, of which compound **18** displayed the best potency against *R. solani*. For instance, after seven days transplantation, the protective and curative activities of **18** (81.16% and 81.58% at 200 μg/mL; 73.66% and 75.59% at 100 μg/mL) and **19** (79.44% and 79.01% at 200 μg/mL; 65.10% and 68.31% at 100 μg/mL) in vivo were more active than those of validamycin A (78.80% and 75.80% at 200 μg/mL; 65.74% and 67.45% at 100 μg/mL).

### 2.3. Preliminary Mode of Action of Compound **18** against R. solani

#### 2.3.1. Effect of **18** on the hyphae morphology of *R. solani*

The untreated *R. solani* mycelia grew smoothly with a low density, and the colony possessed a round and regular edge. However, after treatment with **18**, the edge of the colony became irregular and the mycelia was dense compared to the control mycelia, indicating that **18** seriously inhibited the growth of mycelia.

**Figure 5:**
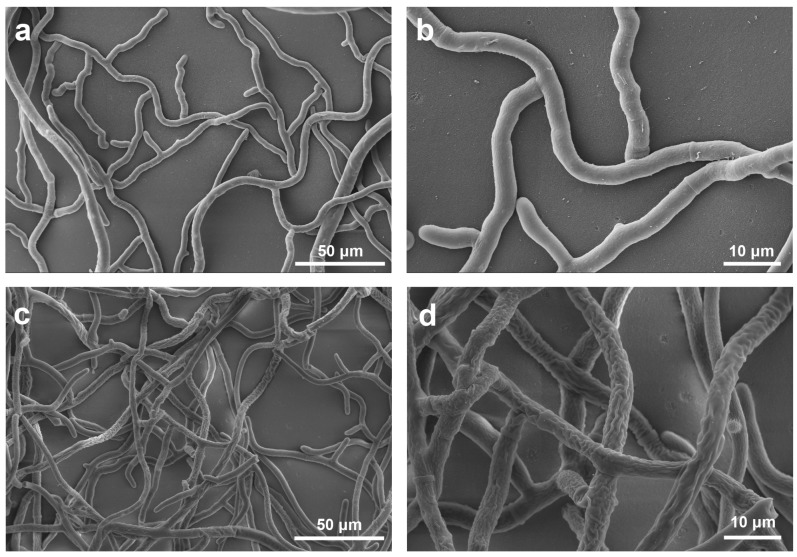
SEM of *R. solani* hyphae treated with 0 (**a**,**b**) or 50 μg/mL **18** (**c**,**d**).

Scanning electron microscopy (SEM) was used to reveal the morphological variations of *R. solani* hyphae in response to **18**. As shown in Figure 5a,b, the mycelia were loose and plump with a smooth surface and an intact structure in the absence of **18**. In contrast, after treatment with 50 μg/mL **18** for 48 h, the mycelia were dense with a coarse surface and appeared severely shrunken and distorted with locally folding and entangling (Figure 5c,d). This prominent morphological change might have resulted from the destruction of cellular organelles.

**Figure 6:**
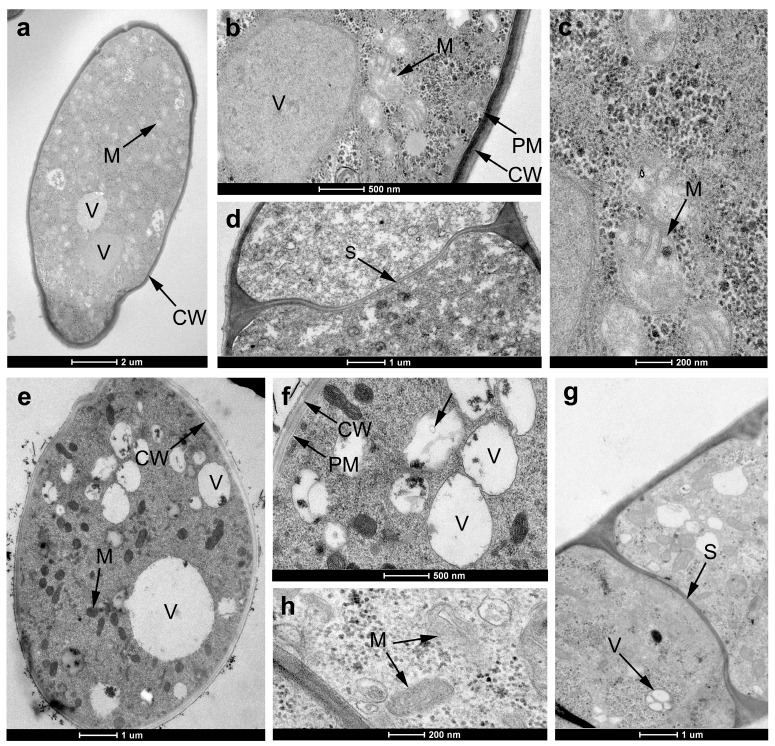
TEM of *R. solani* hyphae treated with 0 (**a**–**d**) or 50 μg/mL **18** (**e**–**h**). (**a**) Transverse of control hyphae and many organelles were observed such as mitochondria (M) and vacuole (V); (**b**) cell wall (CW) and plasma membrane (PM) of untreated hyphae; (**c**) mitochondria of untreated hyphae; (**d**) longitudinal of untreated hyphae, and spectra (S) was uniform; (**e**,**f**) transverse of **18**-treated hyphae; (**g**) longitudinal of **18**-treated hyphae (loss of matrix in vacuoles and obvious vacuolization); (**h**) mitochondria of **18**-treated hyphae was swollen.

Transmission electron microscopy (TEM) observation revealed that the untreated hyphal cell displayed the characteristic ultrastructural features with distinct cell membranes (CMs), cell walls (CWs), and septa (S), and abundant organelles in cytoplasm, such as vacuole (V) and mitochondria (M) (Figure 6a–d). However, after exposure to **18**, the organelles became disorganized and caused profound changes. For example, a slight swelling of the mitochondrial matrix, disappearance of mitochondrial intermembrane space, and an obvious vacuolization were observed (Figure 6e,f,h). While the cell walls and septa of the **18**-treated hyphae were almost unaffected (Figure 6f,g).

#### 2.3.2. Effect of **18** on the Endogenous ROS Production and Cell Membrane Permeability

**Figure 7:**
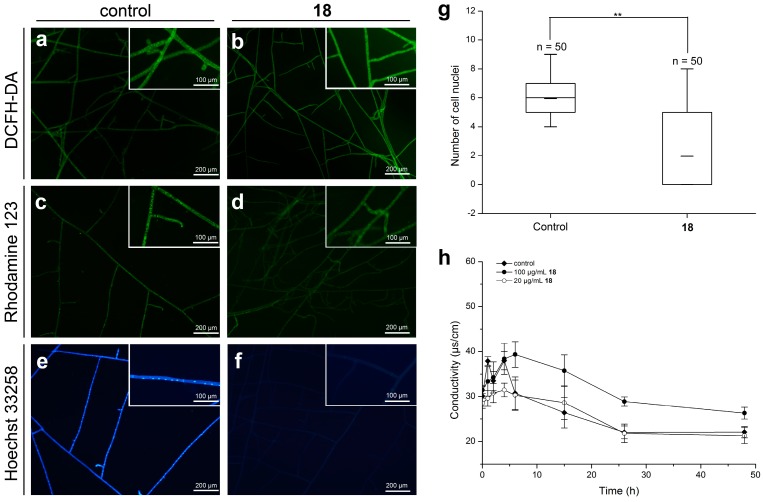
The mode of action of compound **18** against *R. solani*. (**a**,**b**) Fluorescent micrographs of the hyphae stained with DCFH-DA to assess endogenous reactive oxygen species (ROS) production; (**c**,**d**) fluorescent micrographs of the hyphae stained with Rhodamine 123 to evaluate mitochondrial membrane potential (MMP); (**e**,**f**) nuclear morphology of the hyphae stained by Hoechst 33258; (**g**) the number of nuclei per cell of somatic hyphae treated with 0 or 50 μg/mL **18**; (** *p* < 0.01); (**h**) the conductivity of the hyphae suspensions during different time exposure to **18** was measured to assess cell membrane permeability.

Reactive oxygen species (ROS) plays an important role in cell death and involves fungicidal action [18]. To examine the effect of **18** on the intracellular ROS production, an ROS assay was performed on *R. solani* using DCFH-DA (2′,7′-Dichlorodihydrofluorescein diacetate) as an ROS indicator. Interestingly, the significant fluorescence of the **18**-treated mycelia was obviously observed compared to that of the untreated mycelia (Figure 7a,b), which parallels our previous findings for *β*-carboline derivatives [14]. Reactive oxygen species accumulation might induce permeabilization of fungal cell membranes through disintegrating the phospholipid residues [19]. Therefore, we next evaluated the conductivity changes of the hyphae to reflect alterations in cell membrane permeability, and expectedly, the results implied that **18** had a weak effect on cell membrane permeability (Figure 7h), which was similar to what we previously described [14].

#### 2.3.3. Effect of **18** on the mitochondrial membrane potential (MMP)

As an indicator of the energetic atate of the mitochondria, MMP is used to evaluate the activity of the mitochondrial proton pumps, electrogenic transport systems, and the activation of the mitochondrial permeability transition [20]. The effect of **18** on MMP was elucidated using the potential-dependent distributional probe Rhodamine 123. As shown in Figure 7c,d, compared to the control hyphae, a dramatic decrease of fluorescence intensity in the **18**-treated hyphae was observed. This finding suggested that **18** could induce the loss of mitochondria dysfunction, and the subsequent dysfunction of mitochondria, thereby accelerating cell death.

#### 2.3.4. Effect of **18** on the Nuclear Morphology of *R. solani*

The nuclear morphology of *R. solani* hyphae in response to **18** was observed using Hoechst 33258. The results presented in Figure 7e,f showed that the nucleus of the untreated hyphal cells displayed uniform blue color and could be observed as intense, discrete signals. However, after treatment of **18**, the nucleus showed faint signals, and most of the hyphae did not show discrete nuclear signals. Meanwhile, a quantitative analysis was performed to detect the number of nuclei per hyphal cell with or without **18** treatment. As shown in Figure 7g, a dramatic drop in the number of nuclei per **18**-treated hyphal cell was observed, with an average of 1.96 nuclei per cell, compared to that in untreated hyphae (average 5.94 nuclei). Similar results were seen in our previous work [14]. Taken together, these results indicated that compound **18** could interfere with DNA synthesis by reducing DNA contents and the number of cell nuclei.

## 3. Materials and Methods

### 3.1. Chemicals

All reagents and solvents were of reagent grade or purified according to standard methods [21] before use. Reactions were monitored by thin-layer chromatography with silica gel plates using silica gel 60 GF254 (Qingdao Haiyang Chemical Co., Ltd., Qingdao, China). The ^1^H-NMR spectra were recorded on a Bruker Avance-600 superconducting nuclear magnetic resonance instrument (Bruker Company, Bremen, Gemany). The chemical synthesis and structural characterization of compounds **9**–**39** are shown in the Supplementary Materials.

### 3.2. Biological Assay

#### 3.2.1. In Vitro Fungicidal Activity against *R. solani*

The test fungi *R. solani* GD-118 was provided by Zhou of the Guangdong Province Key Laboratory of Microbial Signals and Disease Control, South China Agricultural University. The in vitro fungicidal activity of compounds **1** and **6**–**39** were evaluated according to our previously reported method with validamycin A as a positive control [13].

#### 3.2.2. In Vivo Protective Activity against *R. solani* using Detached Leaf Assay

The detached leaf assay was performed to assess the in vivo protective activity of selected compounds using our previously reported method [13]. Healthy paddy leaves were cut (10 cm in length), sprayed with compounds (200 and 100 μg/mL), and then inoculated with strain *R. solani* after dryness. The efficacy of disease control was calculated by the following formula after cultivation at 25 °C for 5 days.

(1) $$\mathrm{Protective}\;\mathrm{efficacy}\;(\%)=\frac{A_{0}-A_{1}}{A_{0}}\times 100$$

where A_0_ is the diameter of the negative control, and A_1_ is the diameter of the lesion after treatment.

#### 3.2.3. In Vivo Protective and Curative Activities against *R. solani* using Greenhouse Experiment

The rice cultivar (*Xiangyazhan*) was planted following our previously reported method [14]. The plants were sprayed with compounds **17**–**19** (200 and 100 μg/mL) and subsequently cultivated at 25 °C for 24 h before inoculation with *R. solani* by placing a fungus disc (0.6 cm in diameter) on the leaf sheaf of each plant. However, for the curative activity, the rice plants were sprayed with compounds (200 and 100 μg/mL) after inoculation with *R. solani* for 24 h. Validamycin A was used as a positive control, and each treatment was replicated for 20 plants. The diameters of symptoms were measured after inoculation for 7 days and the efficacy of disease control was calculated.

### 3.3. Scanning Electron Microscopy (SEM)

After treatment with 50 μg/mL **18** for 48 h, *R. solani* mycelia tips (0.5 cm) were cut, treated with 2.5% of glutaraldehyde at 4 °C, and subsequently fixed with 1% (*w*/*v*) osmium tetraoxide solution after being rinsed with 0.1 M phosphate buffer (PBS, pH = 7.4) three times. The samples were dehydrated using a series of ethanol solutions (30%, 50%, 70%, 80%, 90%, and 100%), dried at critical point, mounted, gold-sprayed, and then observed under a XL-30-ESEM scanning electron microscope (FEI, Eindhoven, Netherlands) [22].

### 3.4. Transmission Electron Microscopy (TEM)

The dehydrated mycelial blocks were cut into thin sections and then double-stained with uranyl acetate and lead citrate after being embedded in resin, and the samples were observed with a TECNAI G^2^ 12 (FEI) transmission electron microscope.

### 3.5. Determination of ROS Generation

The DCFH-DA staining was used to detect the endogenous ROS generation as described previously [19]. Briefly, after being treated with 50 μg/mL **18** for 48 h, *R. solani* mycelia tips (0.6 cm) were cut and then placed on a sterile slide in a 9-cm culture dish before continued incubation at 25 °C for 24 h. The PDA medium was removed, and the hyphae were stained with 10 μM DCFH-DA solution (Beyotime, Shanghai, China) and incubated at 37 °C for 30 min in the darkness. The samples were observed using a fluorescence microscopy (Nikon ECLIPES 80 i, Tokyo, Japan).

### 3.6. Determination of MMP

The effect of compound **18** on the MMP of *R. solani* was evaluated using Rhodamine 123 staining [14]. The hyphae were stained with 1 mL of 1 μM Rhodamine 123 solution (Beyotime) and then incubated at 37 °C for 30 min in the darkness. The samples were observed under a fluorescence microscopy.

### 3.7. Karyological Analysis

The hyphae were treated with a stain-fixative at 4 °C overnight, and then stained with 1 mL Hoechst 33258 solution (Beyotime) at 25 °C for 10 min after being washed twice with 0.1 M PBS. The samples were observed under a fluorescence microscopy [23].

### 3.8. Detection of Cell Membrane Permeability 

The conductivity changes of *R. solani* mycelial exposed to **18** were evaluated to reflect alterations in cell membrane permeability according to our previously reported method [14].

## 4. Conclusions

In conclusion, optimization of ring C of natural *β*-carboline alkaloids obtained a series of 1,2,4,9-tetrahydro-3-thia-9-aza-fluorene derivatives and their fungicidal activity and preliminary mode of action against *R. solani* were also investigated. The results showed that compound **18** displayed the most activity with an EC_50_ value of 2.35 μg/mL, and was approximately 80-fold more potent than validamycin A. In vivo testing also indicated that **18** could effectively control rice sheath blight caused by *R. solani* and showed better protective and curative activities than validamycin A. Although the preliminary mechanistic study demonstrated that compound **18** might act on the mitochondria, induce loss of MMP and accumulation of ROS, and interfere with DNA synthesis that exerted its fungicidal activity, the specific biological target or targets of **18** remain unknown. Further studies on structural optimization and target identification are in progress.

## Figures and Tables

**Table 1:** In vitro fungicidal activity of compounds **1**, **3**, and **5**–**13**. ^a^

Compound	Percentage Inhibition (%)		EC_50_ (μg/mL)	95% CI ^b^
	100 μg/mL	10 μg/mL		
harmine (**1**)	30.25 ± 1.11	18.28 ± 0.76	318.56	255.10–441.98
harmaline (**3**)	29.51 ± 1.81	14.45 ± 3.76	- ^c^	- ^c^
tetrahydroharmine (**5**)	28.31 ± 6.84	9.03 ± 1.04	- ^c^	- ^c^
*β*-carboline (**6**)	34.04 ± 1.21	16.67 ± 1.34	241.52	109.44–303.76
carbazole (**7**)	56.67 ± 1.69	26.67 ± 2.21	- ^c^	- ^c^
tetrahydro-*β*-carboline (**8**)	5.37 ± 5.45	0	- ^c^	- ^c^
tetrahydro-*γ*-carboline (**9**)	63.95 ± 1.33	10.11 ± 0.08	79.22	71.08–89.41
**10**	69.66 ± 1.21	22.47 ± 1.01	66.89	38.42–94.17
**11**	**91.02 ± 2.31**	**32.45 ± 3.87**	**38.58**	**25.45–51.97**
**12**	3.67 ± 1.69	0.00	>1000	- ^c^
**13**	6.35 ± 1.54	0.00	>1000	- ^c^
validamycin A	36.68 ± 1.09	18.91 ± 0.49	183.00	162.62–210.66

^a^ Values are the mean ± SD of three replicates. ^b^ 95% confidence interval. ^c^ not calculated.

**Table 2:** In vitro fungicidal activity of compounds **14**–**39**. ^a^

Compound	Percentage Inhibition (%)		EC_50_ (μg/mL)	95% CI ^c^
	100 μg/mL	10 μg/mL		
**14**	68.27 ± 1.37	48.05 ± 2.10	37.02	15.40–52.18
**15**	90.80 ± 0.80	57.24 ± 2.76	10.55	0.10–19.73
**16**	89.88 ± 0.80	66.89 ± 2.38	6.84	1.91–13.04
**17**	**93.56 ± 0.80**	**81.61 ± 2.11**	**2.94**	**0.23–6.34**
**18** ^b^	**88.38 ± 0.87**	**82.83 ± 0.87**	**2.35**	**0.42–5.72**
**19**	**89.89 ± 1.33**	**72.15 ± 3.08**	**3.79**	**0.19–7.60**
**20** ^b^	75.53 ± 0.73	55.27 ± 2.64	10.86	2.41–24.47
**21**	68.54 ± 1.36	37.09 ± 1.33	40.15	16.41–71.36
**22**	8.44 ± 3.65	0.00	- ^d^	- ^d^
**23** ^b^	0.00	0.00	- ^d^	- ^d^
**24**	79.77 ± 1.69	43.82 ± 1.33	26.69	14.50–37.00
**25** ^b^	91.92 ± 1.75	52.53 ± 1.75	14.85	1.15–25.21
**26** ^b^	83.84 ± 3.15	40.91 ± 1.52	22.81	9.56–33.56
**27** ^b^	84.85 ± 0.00	59.09 ± 1.51	7.46	4.50–11.21
**28** ^b^	64.65 ± 0.87	28.28 ± 2.31	54.55	39.01–74.92
**29**	75.39 ± 1.21	36.18 ± 1.34	50.93	29.23–83.72
**30** ^b^	50.65 ± 2.25	42.85 ± 1.33	93.08	34.27–172.92
**31** ^b^	67.41 ± 1.69	38.20 ± 2.33	40.38	11.35–56.72
**32** ^b^	50.66 ± 2.97	20.21 ± 0.08	93.42	56.84–372.27
**33** ^b^	87.64 ± 1.63	49.43 ± 3.72	25.65	6.42–46.82
**34**	56.18 ± 1.69	38.20 ± 1.21	67.85	36.64–140.78
**35** ^b^	56.18 ± 1.33	29.21 ± 0.87	83.84	64.84–122.16
**36** ^b^	4.12 ± 0.71	0.00	- ^d^	- ^d^
**37** ^b^	6.33 ± 1.21	0.00	- ^d^	- ^d^
**38** ^b^	37.37 ± 0.74	4.12 ± 0.91	120.49	110.03–135.48
**39** ^b^	38.82 ± 1.47	21.94 ± 3.18	149.44	117.28–226.41

^a^ Values are the mean ± SD of three replicates. ^b^ novel compound. ^c^ 95% confidence interval. ^d^ not calculated.

**Table 3:** In vivo protective activity against *Rhizoctonia solani* using detached leaf assay.

Treatment	Concentration (μg/mL)	Lesion Length ^a^ (cm ± SE)	Control Efficacy (%)
**11**	200	0.61 ± 0.40 **	92.47
	100	1.12 ± 0.45 **	86.17
**14**	200	0.77 ± 0.38 **	90.49
	100	1.69 ± 0.55 **	79.14
**15**	200	0.29 ± 0.19 **	96.42
	100	1.31 ± 0.56 **	83.83
**16**	200	0.17 ± 0.17 **	97.90
	100	0.90 ± 0.45 **	88.89
**17**	200	0 **	**100**
	100	0.21 ± 0.24 **	97.41
**18**	200	0 **	**100**
	100	0.01 ± 0.05 **	99.87
**19**	200	0 **	**100**
	100	0 **	**100**
**21**	200	0.71 ± 0.31 **	91.23
	100	1.69 ± 0.46 **	79.14
validamycin A	200	0 **	100
	100	0 **	100
control	0	8.10 ± 1.43	- ^b^

^**^ represents *p* < 0.01. ^a^ Values are the mean ± SD of 20 leaves. ^b^ not calculated.

**Table 4:** In vivo protective and curative effects against *R. solani* using greenhouse experiment.

Treatment	Concentration (μg/mL)	Protective Effect		Curative Effect	
		Lesion Length ^a^ (cm ± SE)	Control Efficacy (%)	Lesion Length (cm ± SE)	Control Efficacy (%)
**17**	200	0.92 ± 0.59 **	**80.30**	0.99± 0.33 **	**78.80**
	100	1.55 ± 0.73 **	**66.81**	1.76 ± 0.88 **	62.31
**18**	200	0.88 ± 0.47 **	**81.16**	0.86 ± 0.33 **	**81.58**
	100	1.23 ± 0.61 **	**73.66**	1.14 ± 0.39 **	**75.59**
**19**	200	0.96 ± 0.50 **	**79.44**	0.98 ± 0.40 **	**79.01**
	100	1.63 ± 0.45 **	65.10	**1.48** ± 0.50 **	**68.31**
validamycin A	200	0.99 ± 0.43 **	78.80	1.13 ± 0.51 **	75.80
	100	1.60 ± 0.60 **	65.74	1.52 ± 0.64^**^	67.45
control	0	4.67 ± 0.97		4.67 ± 0.97	

** represents *p* < 0.01. ^a^ Values are the mean ± SD of 20 plants.

## Supplementary Materials

Supplementary materials can be found at http://www.mdpi.com/1422-0067/19/12/4044/s1.
