# Supplementary material for: Evolution from Natural β-Carboline Alkaloids to Obtain 1,2,4,9-tetrahydro-3-thia-9-aza-fluorene Derivatives as Potent Fungicidal Agents against Rhizoctonia solani
Source: Int J Mol Sci. 2018 Dec 14;19(12):4044. doi: 10.3390/ijms19124044 (PMC6320924; doi:10.3390/ijms19124044)
Supplement: Supplementary file 1 [file ijms-19-04044-s001.pdf]

Supplementary data

Evolution from natural *β*-carboline alkoloids to obtain 1,2,4,9-tetrahydro-3-thia-9-aza-fluorene derivatives as potent Fungicidal agents against *Rhizoctonia solani*

Junmin Xi,[1] Zhijun Zhang,[1] Qi Zhu, Guohua Zhong[*]

1. General

Compounds **1**, **3**, **5**, **7** and **8** were purchased from BePharm Co., Ltd. (Shanghai, China). Compounds **6** and **10** were prepared according to the procedure reported previously.[1,2] All other reagents and solvents used in the study were analytical grade and obtained from commercial sources.

2. Chemistry

2.1 Synthesis of 2,3,4,5-tetrahydro-1*H*-pyrido[4,3-*b*]indole (9). The mixture of phenylhydrazine hydrochloride (10 mmol) and 1-carbethoxy-4-piperidone (7.66 g, 10 mmol) in absolute EtOH (10 mL) was stirred for 3 h under reflux. The intermediate **40** was filtered and recrystallized from 95% EtOH. To the solution of the intermediate **40** (g, 3.82 mmol) in 15 mL ethanol was added an aqueous solution of KOH (4.28 g,76.4 mmol, in 5 mL water) and the reaction was refluxed for 24 h and concentrated in vacuo. The residual mixture was extracted with DCM. The combined DCM layer was sequentially washed with water and brine, dried, filtered, and concentrated under

reduced pressure to give a brown residue which was purified by silica gel column chromatography (DCM: methanol 25:1) to give **9** as a yellow solid. Yield: 89%; ^{1}H-NMR (400 MHz, $CDCl_3$) δ: 7.46 (d, J = 7.3 Hz, 1H, 6-H), 7.31 (d, J = 8.0 Hz, 1H, 9-H), 7.19-7.10 (m, 2H, 7, 8-H), 4.08 (s, 2H, 1-CH_2-), 3.24 (t, J = 5.8 Hz, 2H, 3-CH_2-), 2.78 (t, J = 5.6 Hz, 2H, 4-CH_2-); anal. calcd for $C_{11}H_{12}N_2$: C, 76.71; H, 7.02; N, 16.27; found: C, 76.59; H, 7.03; N, 16.17.

2.2 General procedure for the synthesis of compounds 11 and 14-28. To a solution of phenyl hydrazine hydrochloride (10 mmol) and tetrahydrothiopyran-4-one (10 mmol) in methanol (40 mL) was added $Bi(NO_3)_3 \cdot 5H_2O$ (970 mg, 2 mmol), and the reaction mixture was stirred for 2 h under reflux, and then poured into water (100 mL). After filtration, the crude product was extracted with ethyl acetate, washed with saturated sodium bicarbonate, dried and concentrated, which was purified by flash chromatography on silica gel with a mixture eluent of petroleum ether and ethyl acetate to give the desired compounds **11** and **14-28**.

1,2,4,9-tetrahydro-3-thia-9-aza-fluorene (11), light yellow solid, yield: 82%; ^{1}H NMR (600 MHz, $CDCl_3$) δ: 7.79 (s, 1H, 5-NH), 7.45 (d, J = 7.8 Hz, 1H, 6-H), 7.30 (d, J = 8.0 Hz, 1H, 9-H), 7.15 (t, J = 7.5 Hz, 1H, 8-H), 7.10 (t, J = 7.4 Hz, 1H, 7-H), 3.87 (s, 2H, 1-CH_2-), 3.02 (s, 4H, 3, 4-CH_2-); anal. calcd for $C_{11}H_{11}NS$: C, 69.80; H, 5.86; N, 7.40; found: C, 69.61; H, 5.87; N, 7.22.

6-methyl-1,2,4,9-tetrahydro-3-thia-9-aza-fluorene (14), light yellow solid, yield: 86%; ^{1}H NMR (600 MHz, $CDCl_3$) δ: 7.69 (s, 1H, 5-NH), 7.23 (s, 1H, 9-H), 7.18 (d, J = 8.2 Hz, 1H, 6-H), 6.97 (d, J = 8.1 Hz, 1H, 7-H), 3.84 (s, 2H, 1-CH_2-),

3.00 (s, 4H, 3, 4-CH_2-), 2.44 (s, 3H, 8-CH_3); anal. calcd for $C_{12}H_{13}NS$: C, 70.89; H, 6.45; N, 6.89; found: C, 70.73; H, 6.44; N, 6.72.

6-fluoro-1,2,4,9-tetrahydro-3-thia-9-aza-fluorene (15), light yellow solid, yield: 89%; ^{1}H NMR (600 MHz, $CDCl_3$) δ: 7.79 (s, 1H, 5-NH), 7.19 (dd, J = 8.7, 4.3 Hz, 1H, 9-H), 7.09 (dd, J = 9.4, 2.3 Hz, 1H, 6-H), 6.88 (td, J = 9.1, 2.4 Hz, 1H, 7-H), 3.81 (s, 2H, 1-CH_2-), 3.01 (s, 4H, 3, 4-CH_2-); anal. calcd for $C_{11}H_{10}FNS$: C, 63.74; H, 4.86; N, 6.76; found: C, 63.58; H, 4.86; N, 6.59.

6-chloro-1,2,4,9-tetrahydro-3-thia-9-aza-fluorene (16), light yellow solid, yield: 92%; ^{1}H NMR (600 MHz, $CDCl_3$) δ: 7.82 (s, 1H, 5-NH), 7.41 (d, J = 1.7 Hz, 1H, 9-H), 7.19 (d, J = 8.5 Hz, 1H, 6-H), 7.09 (dd, J = 8.5, 1.9 Hz, 1H, 7-H), 3.80 (s, 2H, 1-CH_2-), 3.01 (s, 4H, 3, 4-CH_2-); anal. calcd for $C_{11}H_{10}ClNS$: C, 59.09; H, 4.51; N, 6.26; found: C, 58.87; H, 4.53; N, 6.09.

6-bromo-1,2,4,9-tetrahydro-3-thia-9-aza-fluorene (17), light yellow solid, yield: 72%; ^{1}H NMR (600 MHz, $CDCl_3$) δ: 7.85 (s, 1H, 5-NH), 7.56 (d, J = 1.5 Hz, 1H, 9-H), 7.22 (dd, J = 8.5, 1.8 Hz, 1H, 6-H), 7.15 (d, J = 8.5 Hz, 1H, 7-H), 3.80 (s, 2H, 1-CH_2-), 3.00 (s, 4H, 3, 4-CH_2-); anal. calcd for $C_{11}H_{10}BrNS$: C, 49.27; H, 3.76; N, 5.22; found: C, 49.14; H, 3.77; N, 5.06.

6-trifluoromethyl-1,2,4,9-tetrahydro-3-thia-9-aza-fluorene (18), light yellow solid, yield: 38%; ^{1}H NMR (600 MHz, $CDCl_3$) δ: 8.03 (s, 1H, 5-NH), 7.73 (s, 1H, 9-H), 7.39 (d, J = 8.4 Hz, 1H, 6-H), 7.35 (d, J = 8.5 Hz, 1H, 7-H), 3.87 (s, 2H, 1-CH_2-), 3.07-3.02 (m, 4H, 3, 4-CH_2-); anal. calcd for $C_{12}H_{10}F_3NS$: C, 56.02; H, 3.92; N, 5.44; found: C, 55.91; H, 3.91; N, 5.26.

6-nitro-1,2,4,9-tetrahydro-3-thia-9-aza-fluorene (19), light yellow solid, yield: 32%;^{1}H NMR (600 MHz, $CDCl_3$) *δ*: 8.43 (d, *J* = 1.9 Hz, 1H, 6-H), 8.25 (s, 1H, 9-H), 7.32 (d, *J* = 8.9 Hz, 1H, 7-H), 3.88 (s, 2H, 1-CH_2-), 3.07-3.02 (m, 4H, 3, 4-CH_2-); anal. calcd for $C_{11}H_{10}N_2O_2S$: C, 56.39; H, 4.30; N, 11.96; found: C, 56.27; H, 4.31; N, 11.83.

6-trifluoromethoxy-1,2,4,9-tetrahydro-3-thia-9-aza-fluorene (20), light yellow solid, yield: 85%; ^{1}H NMR (600 MHz, $CDCl_3$) *δ*: 7.93 (s, 1H, 5-NH), 7.31 (s, 1H, 9-H), 7.28 (d, *J* = 3.1 Hz, 1H, 6-H), 7.04 (d, *J* = 8.6 Hz, 1H, 7-H), 3.85 (s, 2H, 1-CH_2-), 3.04 (s, 4H, 3, 4-CH_2-); anal. calcd for $C_{12}H_{10}F_3NOS$: C, 52.74; H, 3.69; N, 5.13; found: C, 52.57; H, 3.70; N, 5.02.

6-methoxy-1,2,4,9-tetrahydro-3-thia-9-aza-fluorene (21), light yellow solid, yield: 87%; ^{1}H NMR (600 MHz, $CDCl_3$) *δ*: 7.69 (s, 1H, 5-NH), 7.18 (d, *J* = 8.7 Hz, 1H, 9-H), 6.90 (d, *J* = 2.2 Hz, 1H, 6-H), 6.80 (dd, *J* = 8.7, 2.4 Hz, 1H, 7-H), 3.85 (s, 3H, 8-OCH_3), 3.83 (s, 2H, 1-CH_2-), 3.00 (s, 4H, 3, 4-CH_2-); anal. calcd for $C_{12}H_{13}NOS$: C, 65.72; H, 5.97; N, 6.39; found: C, 65.54; H, 5.93; N, 6.27.

6-benzoxy-1,2,4,9-tetrahydro-3-thia-9-aza-fluorene (22), light yellow solid, yield: 82%; ^{1}H NMR (600 MHz, $CDCl_3$) *δ*: 7.68 (s, 1H, 5-NH), 7.48 (d, *J* = 7.5 Hz, 2H, Ar-H), 7.39 (t, *J* = 7.6 Hz, 2H, Ar-H), 7.32 (t, *J* = 7.3 Hz, 1H, Ar-H), 7.19 (d, *J* = 8.7 Hz, 1H, 9-H), 6.99 (d, *J* = 2.1 Hz, 1H, 6-H), 6.88 (dd, *J* = 8.7, 2.3 Hz, 1H, 7-H), 5.10 (s, 2H, Ph$\underline{CH_2}$O-), 3.82 (s, 2H, 1-CH_2-), 3.00 (s, 4H, 3, 4-CH_2-); anal. calcd for $C_{18}H_{17}NOS$: C, 73.19; H, 5.80; N, 4.74; found: C, 73.04; H, 5.79; N, 4.68.

***N*-(1,2,4,9-tetrahydro-3-thia-9-aza-fluorene-6ylmethyl) methanesulfonamide**

(23), yellow solid, yield: 63%; ^{1}H NMR (600 MHz, $CDCl_3$) δ: 7.89 (s, 1H, 5-NH), 7.46 (s, 1H, 9H), 7.30 (d, J = 8.3 Hz, 1H, 6-H), 7.17 (d, J = 8.1 Hz, 1H, 7-H), 4.36 (s, 2H, -$\underline{CH_2}$-$NHSO_2$-), 3.86 (s, 2H, 1-CH_2-), 3.04-3.00 (m, 4H, 3, 4-CH_2-), 2.70 (d, J = 5.2 Hz, 3H, $\underline{CH_3}SO_2$-); anal. calcd for $C_{13}H_{16}N_2O_2S_2$: C, 52.68; H, 5.44; N, 9.45; found: C, 52.54; H, 5.45; N, 9.31.

Ethyl 1,2,4,9-tetrahydro-3-thia-9-aza-fluorene-6-carboxylate (24), yellow solid, yield: 52%; ^{1}H NMR (600 MHz, $CDCl_3$) δ: 8.22 (s, 1H, 9-H), 8.02 (s, 1H, 5-NH), 7.88 (dd, J = 8.5, 1.5 Hz, 1H, 6-H), 7.31-7.27 (m, 1H, 7-H), 4.40 (q, J = 7.1 Hz, 2H, $CH_3\underline{CH_2}O$-), 3.89 (s, 2H, 4-CH_2-), 3.03 (s, 4H, 3, 4-CH_2-), 1.42 (t, J = 7.1 Hz, 3H, $\underline{CH_3}CH_2O$-); anal. calcd for $C_{14}H_{15}NO_2S$: C, 64.34; H, 5.79; N, 5.36; found: C, 64.19; H, 5.80; N, 5.27.

5-trifluoromethyl-1,2,4,9-tetrahydro-3-thia-9-aza-fluorene (25), yellow solid, yield: 43%; ^{1}H NMR (600 MHz, $CDCl_3$) δ: 8.10 (s, 1H, 5-H), 7.48 (d, J = 8.1 Hz, 1H, 8-H), 7.44 (d, J = 7.6 Hz, 1H, 6-H), 7.18 (t, J = 7.8 Hz, 1H, 7-H), 3.97 (s, 2H, 1-CH_2-), 3.11 (t, J = 5.8 Hz, 2H, 3-CH_2-), 2.99 (t, J = 5.9 Hz, 2H, 4-CH_2-); anal. calcd for $C_{12}H_{10}F_3NS$: C, 56.02; H, 3.92; N, 5.44; found: C, 55.89; H, 3.92; N, 5.28.

7-trifluoromethyl-1,2,4,9-tetrahydro-3-thia-9-aza-fluorene (26), yellow solid, yield: 37%; ^{1}H NMR (600 MHz, $CDCl_3$) δ: 8.02 (s, 1H, 5-NH), 7.57 (s, 1H, 6-H), 7.52 (d, J = 8.2 Hz, 1H, 9-H), 7.34 (d, J = 7.7 Hz, 1H, 7-H), 3.87 (s, 2H, 1-CH_2-), 3.09-3.05 (m, 2H, 3-CH_2-), 3.05-3.01 (m, 2H, 4-CH_2-); anal. calcd for $C_{12}H_{10}F_3NS$: C, 56.02; H, 3.92; N, 5.44; found: C, 55.91; H, 3.91; N, 5.26.

8-trifluoromethyl-1,2,4,9-tetrahydro-3-thia-9-aza-fluorene (27), yellow solid,

yield: 56%; ^{1}H NMR (600 MHz, $CDCl_3$) δ: 8.19 (s, 1H, 5-NH), 7.62 (d, J = 7.9 Hz, 1H, 7-H), 7.40 (d, J = 7.5 Hz, 1H, 9-H), 7.16 (t, J = 7.7 Hz, 1H, 8-H), 3.86 (s, 2H, 1-CH_2-), 3.09-3.05 (m, 2H, 3-CH_2-), 3.05-3.02 (m, 2H, 4-CH_2-); anal. calcd for $C_{12}H_{10}F_3NS$: C, 56.02; H, 3.92; N, 5.44; found: C, 55.94; H, 3.93; N, 5.22.

5,7-di-trifluoromethyl-1,2,4,9-tetrahydro-3-thia-9-aza-fluorene (28), yellow solid, yield: 35%; ^{1}H NMR (600 MHz, $CDCl_3$) δ: 7.37 (s, 1H, 8-H), 7.29 (s, 1H, 6-H), 2.90-2.85 (m, 2H, 1-CH_2-), 2.83-2.80 (m, 2H, 3-CH_2-), 2.78-2.74 (m, 2H, 4-CH_2-); anal. calcd for $C_{13}H_9F_6NS$: C, 48.00; H, 2.79; N, 4.31; found: C, 47.92; H, 2.80; N, 4.25.

2.3 Synthesis of 1,2,4,9-tetrahydro-3-thia-9-aza-fluorene-3-oxide (12). To a solution of **11** (378 mg, 2 mmol) in THF (20 mL) was added $BF_3.Et_2O$ (1.13 g, 8 mmol) at -20 °C under N_2. *m*-Chloroperbenzoic acid was then added (345 mg, 2 mmol) at -20 °C and the mixture was stirred at -20 °C for 2 h, poured into $NaHCO_3$ (30 mL) and subsequently extracted with ethyl acetate (30 mL). The combined organic phase was washed with water, dried and filtrated to obtain the crude sulfoxide **12**, which was purified by flash chromatography. Yield: 73%; ^{1}H NMR (600 MHz, $CDCl_3$) δ: 7.99 (s, 1H, 5-NH), 7.45 (d, J = 7.8 Hz, 1H, 6-H), 7.31 (d, J = 8.1 Hz, 1H, 9-H), 7.19 (t, J = 7.6 Hz, 1H, 8-H), 7.13 (t, J = 7.5 Hz, 1H, 7-H), 4.27 (d, J = 15.0 Hz, 1H, 1-CH_2-), 4.03 (d, J = 15.0 Hz, 1H, 1-CH_2-), 3.40 (tt, J = 13.9, 7.1 Hz, 2H, 3-CH_2-), 3.19-3.11 (m, 2H, 4-CH_2-); anal. calcd for $C_{11}H_{11}NOS$: C, 64.36; H, 5.40; N, 6.82; found: C, 64.23; H, 5.40; N, 6.68.

2.4 Synthesis of 1,2,4,9-tetrahydro-3-thia-9-aza-fluorene-3,3-dioxide (13). To a

solution of **11** (378 mg, 2 mmol) in 20 mL THF was added *m*-chloroperbenzoic acid in THF (5 mmol) at 0 °C. The mixture was stirred at room temperature for 2 h. After concentration, the residue was dissolved in ethyl acetate (30 mL). The solution was washed with Na_2SO_3, water and brine. After filtration and concentration, the residue was purified by flash chromatography to obtain sulfone **13** as a light yellow solid. Yield: 58%; ^{1}H NMR (600 MHz, $CDCl_3$) δ: 7.99 (s, 1H, 5-NH), 7.39 (d, J = 7.8 Hz, 1H, 6-H), 7.33 (d, J = 8.1 Hz, 1H, 9-H), 7.24-7.20 (m, 1H, 8-H), 7.15 (dd, J = 11.0, 4.0 Hz, 1H, 7-H), 4.39 (s, 2H, 1-CH_2-), 3.43 (t, J = 5.9 Hz, 2H, 3-CH_2-), 3.36 (t, J = 6.1 Hz, 2H, 4-CH_2-); anal. calcd for $C_{11}H_{11}NO_2S$: C, 59.71; H, 5.01; N, 6.33; found: C, 59.54; H, 4.99; N, 6.21..

2.5 General procedure for the synthesis of compounds 29-35. To a solution of **11** (189 mg, 1 mmol) in DMF (10 mL) were added sodium hydride (44 mg, 1.1 mmol) at 0 ºC. After stirred at 0 ºC for 30 min, various alkyl halides or acyl chloride (1.1 mmol) was added and the reaction mixture were stirred at room temperature for 2 h. Then the reaction mixture was diluted with water and extracted with ethyl acetate (3×15 mL). The extracts were combined, washed with brine, dried over $MgSO_4$, filtered and concentrated in vacuo. The residue was then purified by flash chromatography on silica gel with a mixture eluent of petroleum ether and ethyl acetate to give the desired compound **29-35**.

***N*-methyl-1,2,4,9-tetrahydro-3-thia-9-aza-fluorene (29)**, white solid, yield: 90%; ^{1}H NMR (600 MHz, $CDCl_3$) δ: 7.47 (d, J = 7.8 Hz, 1H, 9-H), 7.29 (d, J = 8.2 Hz, 1H, 6-H), 7.19 (dd, J = 11.2, 4.0 Hz, 1H, 8-H), 7.10 (t, J = 7.4 Hz, 1H, 7-H), 3.89

(s, 2H, 1-CH_2-), 3.63 (s, 3H, N-CH_3), 3.06 (t, J = 5.6 Hz, 2H, 3-CH_2-), 3.00 (t, J = 5.4 Hz, 2H, 4-CH_2-); anal. calcd for $C_{12}H_{13}NS$: C, 70.89; H, 6.45; N, 6.89; found: C, 70.72; H, 6.45; N, 6.77.

***N*-ethoxycarbonyl-1,2,4,9-tetrahydro-3-thia-9-aza-fluorene (30)**, white solid, yield: 85%; ^{1}H NMR (600 MHz, $CDCl_3$) δ: 8.12 (d, J = 8.2 Hz, 1H, 9-H), 7.40 (d, J = 7.6 Hz, 1H, 6-H), 7.28 (dd, J = 11.3, 4.1 Hz, 1H, 8-H), 7.24 (d, J = 7.2 Hz, 1H, 7-H), 4.49 (q, J = 7.1 Hz, 2H, $CH_3\underline{CH_2}O$-), 3.79 (s, 2H, 1-CH_2-), 3.32 (t, J = 5.8 Hz, 2H, 3-CH_2-), 2.99 (t, J = 5.9 Hz, 2H, 4-CH_2-), 1.49 (t, J = 7.1 Hz, 3H, $\underline{CH_3}CH_2O$-); anal. calcd for $C_{14}H_{15}NO_2S$: C, 64.34; H, 5.79; N, 5.36; found: C, 64.18; H, 5.81; N, 5.27.

Methyl *N*-1,2,4,9-tetrahydro-3-thia-9-aza-fluorene acetate (31), white solid, yield: 77%; ^{1}H NMR (600 MHz, $CDCl_3$) δ: 7.48 (d, J = 7.8 Hz, 1H, 9-H), 7.22-7.17 (m, 2H, 6-H, 8-H), 7.13 (t, J = 7.9 Hz, 1H, 7-H), 4.77 (s, 2H, -$\underline{CH_2}$CO-), 3.89 (s, 2H, 1-CH_2-), 3.74 (s, 3H, $COOCH_3$), 3.05 (t, J = 5.8 Hz, 2H, 3-CH_2-), 2.94 (t, J = 5.7 Hz, 2H, 4-CH_2-); anal. calcd for $C_{14}H_{15}NO_2S$: C, 64.34; H, 5.79; N, 5.36; found: C, 64.19; H, 5.80; N, 5.27.

Ethyl *N*-1,2,4,9-tetrahydro-3-thia-9-aza-fluorene acetate (32), white solid, yield: 81%; ^{1}H NMR (600 MHz, $CDCl_3$) δ: 7.47 (d, J = 7.8 Hz, 1H, 9-H), 7.22-7.17 (m, 2H, 6-H, 8-H), 7.12 (t, J = 7.2 Hz, 1H, 7-H), 4.74 (s, 2H, -$\underline{CH_2}$CO-), 4.20 (q, J = 7.1 Hz, 2H, $CH_3\underline{CH_2}O$-), 3.89 (s, 2H, 1-CH_2-), 3.05 (t, J = 5.8 Hz, 2H, 3-CH_2-), 2.94 (t, J = 5.7 Hz, 2H, 4-CH_2-), 1.26 (t, J = 7.1 Hz, 3H, $\underline{CH_3}CH_2O$-); anal. calcd for $C_{15}H_{17}NO_2S$: C, 65.43; H, 6.22; N, 5.09; found: C, 65.27; H, 6.21; N, 4.97.

Methyl *N*-1,2,4,9-tetrahydro-3-thia-9-aza-fluorene valerate (33), yellow oil,

yield: 73%; ^{1}H NMR (600 MHz, $CDCl_3$) δ: 7.46 (d, J = 7.8 Hz, 1H, 9-H), 7.27 (d, J = 8.2 Hz, 1H, 6-H), 7.17 (t, J = 7.2 Hz, 1H, 8-H), 7.09 (t, J = 7.3 Hz, 1H, 7-H), 4.04 (t, J = 7.3 Hz, 2H, 5-N$\underline{CH_2}$-), 3.88 (s, 2H, 1-CH_2-), 3.66 (s, 3H, $COOCH_3$), 3.05 (t, J = 5.5 Hz, 2H, 3-CH_2-), 2.99 (t, J = 5.2 Hz, 2H, 4-CH_2-), 2.32 (t, J = 7.2 Hz, 2H, -$\underline{CH_2}$COO-), 1.77 (m, 2H, CH_2), 1.70-1.65 (m, 2H, CH_2); anal. calcd for $C_{17}H_{21}NO_2S$: C, 67.29; H, 6.98; N, 4.62; found: C, 67.07; H, 6.99; N, 4.53.

***N*-benzyl-1,2,4,9-tetrahydro-3-thia-9-aza-fluorene valerate (34)**, yellow oil, yield: 71%; ^{1}H NMR (600 MHz, $CDCl_3$) δ: 7.51 (d, J = 7.6 Hz, 1H, 9-H), 7.37 (dt, J = 26.3, 7.5 Hz, 1H, Ar-H), 7.29-7.22 (m, 3H, 6-H, Ar-H), 7.15 (t, J = 7.1 Hz, 1H, 8-H), 7.12 (t, J = 7.1 Hz, 1H, 7-H), 6.98 (d, J = 7.3 Hz, 2H, Ar-H), 5.28 (s, 2H, Ph$\underline{CH_2}$-), 3.93 (s, 2H, 1-CH_2-), 3.01 (t, J = 5.8 Hz, 2H, 3-CH_2-), 2.91 (t, J = 5.7 Hz, 2H, 4-CH_2-); anal. calcd for $C_{18}H_{17}NS$: C, 77.38; H, 6.13; N, 5.01; found: C, 77.23; H, 6.14; N, 4.93.

***N*-[(1,2,4,9-tetrahydro-3-thia-9-aza-fluorene)methyl]phenyl-*β*-methoxy methacrylate (35)**, yellow solid, yield: 82%; ^{1}H NMR (600 MHz, $CDCl_3$) δ: 7.65 (s, 1H, 9-H), 7.50 (dd, J = 5.8, 2.8 Hz, 1H, Ar-H), 7.25-7.21 (m, 2H, Ar-H), 7.16 (d, J = 7.2 Hz, 2H, Ar-H), 7.12-7.09 (m, 3H, Ar-H), 6.45 (d, J = 7.7 Hz, 1H, =$\underline{CH}$O-), 5.12 (s, 2H, CH_2), 3.92 (s, 2H, 1-CH_2-), 3.91 (s, 3H, COO$\underline{CH_3}$), 3.77 (s, 3H, =CHO$\underline{CH_3}$), 2.97 (t, J = 5.8 Hz, 2H, 3-CH_2-), 2.82 (t, J = 5.7 Hz, 2H, 4-CH_2-); anal. calcd for $C_{23}H_{23}NO_3S$: C, 70.20; H, 5.89; N, 3.56; found: C, 70.07; H, 5.90; N, 3.42.

2.6 Synthesis of *N*-1,2,4,9-tetrahydro-3-thia-9-aza-fluorene carbohydrazide (36).

To a solution of compound **11** (378 mg, 2 mmol) in ethanol (10 mL) was added 2 mL

hydrazine hydrate and the mixture was heated under reflux for 6 h. The reaction mixture was then cooled and filtrated to give compound 36 as a light yellow solid, yield: 81%; ^{1}H NMR (600 MHz, $CDCl_3$) *δ*: 7.50 (d, *J* = 7.7 Hz, 1H, 9-H), 7.22 (t, *J* = 8.3 Hz, 2H, 6-H, 8-H), 7.18 (t, *J* = 7.1 Hz, 1H, 7-H), 6.50 (s, 1H, NH), 4.76 (s, 2H, -$\underline{CH_2}$CO-), 3.87 (s, 2H, 1-CH_2-), 3.78 (d, *J* = 3.7 Hz, 2H, NH_2), 3.05 (t, *J* = 5.7 Hz, 2H, 3-CH_2-), 2.91 (t, *J* = 5.5 Hz, 2H, 4-CH_2-); anal. calcd for $C_{13}H_{15}N_3OS$: C, 59.74; H, 5.79; N, 16.08; found: C,59.63; H, 5.78; N, 15.93.

2.7 Synthesis of *N*-(3,4,5-trimethoxybenzylidene)-1,2,4,9-tetrahydro-3-thia-9-aza-fluorene carbohydrazide (37). To a solution of **36** (261 mg, 1 mmol) in absolute ethanol (10 mL) was added dropwise 3,4,5-trimethoxybenzaldehyde (196 mg, 1 mmol) and acetic acid (1 mL), and the reaction mixture was then refluxed for 6 h. After cooled to room temperature, the mixture was filtrated to obtain compound **37** as light yellow solid, yield: 94%; ^{1}H NMR (600 MHz, $CDCl_3$) *δ*: 9.14 (s, 1H, NH), 7.49 (d, *J* = 7.7 Hz, 1H, 9-H), 7.30 (t, *J* = 8.8 Hz, 1H, 6-H), 7.22 (t, *J* = 7.4 Hz, 1H, 8-H), 7.14 (dt, *J* = 32.3, 7.5 Hz, 2H, 7-H), 6.91 (s, 2H, Ar-H), 6.86 (s, 1H, -CH=NN-), 5.27 (s, 2H, CH_2), 4.87 (s, 2H, 1-CH_2-), 3.95 (s, 6H, 3',5'-OCH_3), 3.85 (s, 3H, 4'-OCH_3)), 3.10-3.04 (m, 2H, 3-CH_2-), 3.00 (dd, *J* = 11.9, 5.5 Hz, 2H, 4-CH_2-) ; anal. calcd for $C_{23}H_{25}N_3O_4S$: C, 62.85; H, 5.73; N, 9.56; found: C, 62.72; H, 5.74; N, 9.43.

2.8 Synthesis of *N*-(2-thioxo-1,3,4-oxadiazol-5-yl)-1,2,4,9-tetrahydro-3-thia-9-aza-fluorene (38). To a solution of **36** (261 mg, 1 mmol) in 10 mL absolute ethanol were added carbon disulfide (0.3 mL, 5 mmol) and potassium hydroxide (84 mg, 1.5 mmol) and the mixture was then heated under reflux for 48 h. After concentration, the residue

was diluted with water and acidified with 2N HCl solution. The product **38** was obtained by filtration and recrystallization from methanol. Yield: 73%; ^{1}H NMR (600 MHz, $CDCl_3$) δ: 7.48 (d, J = 7.7 Hz, 1H, 9-H), 7.34 (d, J = 8.1 Hz, 1H, 6-H), 7.23 (d, J = 7.7 Hz, 1H, 8-H), 7.16 (t, J = 7.3 Hz, 1H, 7-H), 5.21 (s, 2H, CH_2), 3.86 (s, 2H, 1-CH_2-), 3.08 (d, J = 3.3 Hz, 4H, 3, 4-CH_2-); anal. calcd for $C_{14}H_{13}N_3OS_2$: C, 55.42; H, 4.32; N, 13.85; found: C,55.27; H, 4.33; N, 13.73.

2.9 Synthesis of *N*-(2-chlorobenzoyl)-1,2,4,9-tetrahydro-3-thia-9-aza-fluorene (39). The compound **39** was synthesized with **11** and 2-chlorobenzoyl chloride using a similar procedure as for compound **29**. Yield: 88%; ^{1}H NMR (600 MHz, $CDCl_3$) δ: 7.54-7.47 (m, 4H, Ar-H, 9-H), 7.44-7.39 (m, 2H, Ar-H, 6-H), 7.26-7.22 (m, 1H, 8-H), 7.15-7.11 (m, 1H, 7-H), 3.82 (s, 2H, 1-CH_2-), 2.89 (t, J = 6.1 Hz, 4H, 3, 4-CH_2-); anal. calcd for $C_{18}H_{14}ClNOS$: C, 65.95; H, 4.30; N, 4.27; found: C,65.77; H, 4.31; N, 4.20.

REFERENCES

1. Su, H.; Wang, W.; Bao, L.; Wang, S.; Cao, X. Synthesis and evaluation of essential oil-derived β-methoxyacrylate derivatives as high potential fungicides. *Molecules* **2017**, *22*, 763, doi:10.3390/molecules22050763.
2. Siddalingamurthy, E.; Mahadevan, K.M.; Masagalli, J.N.; Harishkumar, H.N. Mild, efficient Fischer indole synthesis using 2,4,6-trichloro-1,3,5-triazine (TCT). *Tetrahedron Lett.* **2013**, *54*, 5591-5596, doi:10.1016/j.tetlet.2013.07.157.
